# Stone Mountain Medical Association

**Published:** 1887-08

**Authors:** 


### STONE MOUNTAIN MEDICAL ASSOCIATION.

This Association met at Decatur, on July 7th, being the fourth meeting since its organization. It was called to order by President Word. Dr. J H. Green read an interesting report of a case of puerperal convulsions, which, with an abstract of the discussions thereon, we hope to give in a future number of this journal.

Dr. Lallerstedt inquired as to the method used by members in reviving asphyxiated babes at birth.

Dr. Green and Dr. Jones advocated the usual methods of manipulating and tossing the babe, artificial respiration, hot bathing, etc.

Dr. Lallerstedt said he had found that dipping the infant in hot water and holding it up for a short time by the feet, with the head down, would promptly revive it.

Dr. Word mentioned a recent case of cholera morbus, in which the patient had run down in a few hours' time to an extreme condition of prostration, cramps and collapse. The extremities were cold, he was pulseless and breathed with great difficulty. He at once inserted, hypodermically, ⅓ grain morphine with 1-80th of grain atropia, applied sinapisms to the extremities and to back and abdomen, followed by warmth to the extremities with frictions, and the following remedy internally:

℞. Dilute alcohol........................................℥ ij,
Carbolic acid........................................gtts. ij,
Chloroform........................................gtts. xx.

This was given at a dose, and repeated in twenty-five minutes, by which time the reaction commenced, and in less than one hour the circulation was well restored and the patient in all respects relieved.

Dr. Lallerstedt reported a case somewhat like Dr. Word's case, in which his patient was also relieved with ⅛ grain of morphine hypodermically with atropia, with hot water applications to backbone and alcohol and chloroform internally.

Dr. L. invited the attention of the members to the wonderful powers of gelsemium in fevers and other affections.

"The remedy," he said, "was not appreciated, because the profession in a large degree was ignorant of its powers, and have overestimated its dangers." He had found it a sovereign remedy in the so-called typhoid fevers of the country, and had been able to cut short the disease with gelsemium pushed to its constitutional effect.

Dr. Word said that the announcement made by Dr. L., that our typho-malarial and typhoid fevers could be cut short with gelsemium was a very important one, and would give a world-wide fame to Dr. L. and this Association should it be verified in the experience of the profession.

Dr. L. said he would not say that it would avert typhoid fever, but that he could cure the worst forms of fever he had seen, and that he did not believe there was any genuine typhoid fevers in this country. The remedy, however, must be given in large and oft-repeated doses. He usually gave 20-drop doses repeated every hour until the fever goes off; he continued the remedy as long as the fever lasted. He mentioned a recent obstinate case which had been in the hands of a brother practitioner, on which he commenced with 25 drops of tincture gelsemium and followed in an hour with 20 drops, and the third hour with 15 drops. The fever was entirely off on the day after he had commenced the treatment, notwithstanding the temperature was at 105 and the pulse at 140. He was in the habit of giving large doses of quinine during the remission of fevers and large doses of gelsemium during the hot stage.

He mentioned another case in which the patient had been taking 5-drop doses every two hours. He doubled the dose and gave it every hour, and after five doses the fever gave way.

Dr. Green said he had for some time been using antipyrin during the height of the fever, and quinine during the remission, with good results.

Dr. Hart mentioned a recent case of typhoid fever in his practice, in which was high temperature, dry tongue and tympanitis that seemed to be aborted by a profuse sweating following upon the use of jaborandi.

Dr. Jones said he had relieved a bad sick headache with a dose of 40 drops of gelsemium combined with 60 grains of bromide of potassium.

Dr. Hart mentioned a case of metastasis of mumps to the brain instead of to the testicle. The patient recovered after using a blister to the parotid. Two weeks afterwards another member of the family had inflammation of the brain after seeming to recover from the mumps.

Dr. Word mentioned that in Atlanta, last winter, during the prevalence of mumps and measles, a number of such cases had occurred and proved fatal. They were regarded by some as cases of meningitis. The coincidence of meningitis with the prevalence of mumps, measles and influenzas has been often observed and is mentioned by authors.

Dr. Jones asked if it were true that mumps would affect one side, and after a long interval attack the other side?

Dr. Word said that it was a common opinion that mumps in one parotid gland did not protect from a second attack on the other side, yet theoretically it is unreasonable that a specific, contagious and self-limited disease could be confined in its constitutional impressions to one side of the body. The idea must have resulted from mistaking other affections of the parotid gland for mumps.

